# Effects of mesenchymal stem cells on interleukin-1β-treated chondrocytes and cartilage in a rat osteoarthritic model

**DOI:** 10.3892/mmr.2015.3645

**Published:** 2015-04-20

**Authors:** JILEI TANG, WEIDING CUI, FANGLONG SONG, CHENJUN ZHAI, HANSHENG HU, QIANG ZUO, WEIMIN FAN

**Affiliations:** 1 Department of Orthopedics, The First Affiliated Hospital of Nanjing Medical University, Nanjing, Jiangsu 210029, P.R. China; 2 Department of Orthopedics, Qidong People’s Hospital, Nantong, Jiangsu 226200, P.R. China

**Keywords:** mesenchymal stem cells, chondrocytes, interleukin-1β, osteoarthritis

## Abstract

In the present study, the effects and mechanisms of mesenchymal stem cells (MSCs) on interleukin (IL)-1β-stimulated rat chondrocytes, as well as cartilage from a rat model of osteoarthritis (OA) induced by anterior cruciate ligament transection and medial meniscectomy were investigated. Confluent rat chondrocytes were treated with IL-1β (10 ng/ml), then cultured indirectly with or without MSCs at a ratio of 2:1. Total RNA and protein were collected at various time-points, and western blot and reverse transcription-quantitative polymerase chain reaction analyses were used to investigate the expression of type II collagen (Col2), aggrecan, matrix metalloproteinase-13 (MMP-13) and cyclooxygenase-2 (COX-2). The activation of extracellular signal-regulated kinases 1/2 (ERK1/2), c-Jun N-terminal kinase (JNK), p38 mitogen-activated protein kinase (MAPK), nuclear factor-κB (NF-κB) p65 and inhibitory-κ-B-α (IκBα) were also assessed by western blotting. In addition, the *in vivo* effects of MSCs in a rat OA model were assessed by histology and western blot analysis. The results indicated that *in vitro*, IL-1β markedly upregulated the expression of MMP-13, COX-2, phosphorylated ERK1/2, JNK, p38 MAPK and NF-κB p65, and inhibited the expression of Col2, aggrecan and IκBα. Conversely, MSCs enhanced the expression of Col2, aggrecan and IκBα, and inhibited the expression of MMP-13 and NF-κB p65 in IL-1β-stimulated rat chondrocytes. *In vivo* histological and western blot analyses revealed analogous results to the *in vitro* findings. The results of the present study demonstrated that MSCs suppressed the inflammatory response and extracellular matrix degradation in IL-1β-induced rat chondrocytes, as well as cartilage in a osteoarthritic rat model, in part via the NF-κB signaling pathway.

## Introduction

Osteoarthritis (OA) is a common degenerative and inflammatory joint disease (1). To date, no drugs are available which are able to structurally modify OA processes or prevent progression of the disease (2). Mesenchymal stem cells (MSCs), differ from the single-effect function of drugs and secrete numerous bioactive agents that inhibit inflammation, suppress immune recognition and stimulate host progenitors to divide and differentiate into functional regenerative units (3). MSCs have been widely used in cartilage regeneration therapies (4), and certain animal studies and initial clinical studies have demonstrated the potential of MSC implantation as an alternative treatment for OA (5–10).

Interleukin-1β (IL-1β), one of the most significant pro-inflammatory cytokines involved in OA, diminishes the expression of type II collagen (Col2) and aggrecan through the expression of matrix metalloproteinases (MMPs) and cyclooxygenase-2 (COX-2) (11,12). This effect is mediated by the mitogen-activated protein kinases (MAPKs) and nuclear factor-κB (NF-κB) pathways, which have been described as the link between inflammation and joint cartilage degeneration in OA (13–15).

Despite the implication of MSCs in the regulation of inflammation, little is known regarding the effects of MSCs on IL-1β-stimulated rat chondrocytes and intracellular signaling pathways. The present study therefore aimed to investigate the effects and mechanism of MSCs on IL-1β-treated rat chondrocytes and on cartilage in a rat model of OA induced by anterior cruciate ligament transection and medial meniscectomy. The effects of MSCs on IL-1β-treated chondrocytes and cartilage in this model were evaluated by analyzing the expression of Col2, aggrecan, MMP-13 and COX-2, as well as the activation of extracellular signal-regulated kinases 1/2 (ERK1/2), c-Jun N-terminal kinase (JNK), p38 MAPK and NF-κB pathways.

## Materials and methods

### Ethical approval

All experimental procedures involving animals were conducted in accordance with the National Institutes of Health Guidelines for the Care and Use of Laboratory Animals (www.nap.edu/openbook.php?record_id=5140) and were approved by the Committee for the Administration of Experimental Animals, Nanjing Medical University (Nanjing, China). The experiments were additionally approved by the Animal Ethical And Welfare Committee of Nanjing Medical University.

### Preparation of MSCs

Sprague-Dawley (SD) rat bone marrow MSCs were harvested and cultured as described previously (16). Briefly, the femurs and tibias were dissected away from the attached soft tissue under aseptic conditions and the epiphyses were removed. The bone marrow was flushed out with phosphate-buffered saline (PBS) containing heparin (Gibco Life Technologies, Carlsbad, CA, USA). The mixture was separated by density centrifugation through lymphocyte separation solution (1.073 g/ml; Gibco Life Technologies) at 1000 × g for 15 min at 24°C. Subsequently the mononuclear fraction interphase was collected, resuspended in Dulbecco’s modified Eagle’s medium (DMEM)/F12 (Gibco Life Technologies) supplemented with 10% fetal bovine serum (FBS), 100 U/ml penicillin G and 100 *µ*g/ml streptomycin (all from Gibco Life Technologies) and seeded into culture flasks (cultivation area, 25-cm^2^) containing 5 ml culture medium (DMEM/F12). The cells were cultured at 37°C in 5% CO_2_ for three days, following which the non-adherent cell population was removed. The medium was subsequently changed every 48 h, and cells of passage three were induced to differentiate into adipogenic [DMEM/F12, 10% FBS, 1 *µ*mol/l dexamethasone (DEX), 0.5 mmol/l : 3-isobutyl-1-methylxanthine, 5 mg/l insulin, 100 *µ*mol/l indomethacin; (17)], osteogenic [DMEM/F12, 10% FBS, 100nmol/l DEX, 10 mmol/l β-sodium glycerophosphate, 50 *µ*g/ml vitamin C (Vc); (17)] and chondrogenic [DMEM/F12, 10% FBS and 1% insulin-transferrin-selenium all purchased from Sigma-Aldrich (San Francisco, CA, USA), and 10 ng/ml transforming growth factor (TGF)-β1 (Gibco Life Technologies), 0.1 *µ*mol/l DEX, 50 *µ*g/ml Vc; (18)] lineages.

### Chondrocyte culture and treatment

Chondrocytes were harvested from rat articular cartilage as described previously (19). In brief, under sterile conditions, cartilage tissues derived from the limb joints of 2-week-old SD rats were cut into small sections (<1 mm^3^) and digested with 0.2% trypsin (Gibco Life Technologies) and 0.2% type II collagenase (Gibco Life Technologies) for 30 min and 2 h, respectively. The isolated chondrocytes were resuspended in DMEM/F12 supplemented with 10% FBS, 100 U/ml penicillin G and 100 *µ*g/ml streptomycin. The culture medium was changed every other day. Following passage two, the cells reached ~70–80% confluence and the medium was replaced with DMEM/F12 supplemented with 0.5% FBS and antibiotics for 12 h. Following synchronization, 10 ng/ml IL-1β (R&D Systems, Minneapolis, MN, USA) was added to the culture medium and the cells were cultured for a further 24 h. At the end of this treatment period, the cells were cultured with normal medium for 3, 6 and 12 days (for analysis of Col2 and aggrecan) or for 1, 2 and 4 days (for COX-2 and MMP-13 analysis). Total RNA and protein were collected for reverse transcription-quantitative polymerase chain reaction and western blot analysis. Proteins were extracted from cells by washing chondrocyte monolayers three times with PBS, and extracting whole cell proteins by incubation with lysis buffer (50 mM Tris/HCl pH 7.2, 150 mM NaCl, l% (v/v) Triton X-100, 1 mM sodium orthovanadate, 50 mM sodium pyrophosphate, 100 mM sodium fluoride, 0.01% (v/v) aprotinin, 4 *µ*g/ml pepstatin A, 10 *µ*g/ml leupeptin and 1 mM phenylmethanesulfonyl fluoride, all purchased from Beyotime Institute of Biotechnology, Jiangsu, China) on ice for 30 min and removal of cell debris by centrifugation. The supernatants were then stored at −70°C. Proteins were extracted from cartilage by crushing the tissue, then incubating the tissue with lysis buffer as described above. RNA was collected according to the protocol outlined at www.biomart.cn/experiment/430/443/728/65628.htm. Additionally, following serum starvation, IL-1β (10 ng/ml) was added to the culture medium for 15, 30 or 60 min to investigate ERK1/2, JNK and p38 MAPK signaling, as well as IκBα and NF-κB p65. Controls were cultured in DMEM/F12 with 0.5% FBS without IL-1β. Protein was collected for western blot analysis.

### Co-culture

For co-culture without direct cell-cell contact, passage three MSCs were seeded onto Transwell inserts (six-well plates; BD Biosciences, Franklin Lakes, NJ, USA) with a 0.4-*µ*m porous membrane (BD Biosciences) and lowered into wells seeded with passage two chondrocytes. Following IL-1β stimulation, the chondrocytes were cultured with or without MSCs for three days (for analysis of Col2 and aggrecan) or for one day (for COX-2 and MMP-13 analysis). The number of chondrocytes and MSCs used was 3.0×10^4^ and 1.5×10^4^, respectively, and the ratio was that previously identified as the optimal ratio (19). Total RNA and protein were collected for reverse transcription-quantitative polymerase chain reaction (RT-qPCR) and western blot analysis. In addition, subconfluent monolayers of IL-1β-induced chondrocytes were cultured with or without MSC-conditioned medium (5 ml) for 15 min (for analysis of IκBα and NF-κB p65) or for 30 min (for ERK1/2, JNK and p38 MAPK signaling analysis). The MSCs and IL-1β-induced chondrocytes were co-cultured for 24 h and the medium was collected as MSC-conditioned medium. Protein was collected for western blot analysis. The incubation times were based on previous analysis of cultured chondrocytes by our group (20).

### Animal study

Thirty-six male SD rats (Central Lab Animal Inc., Nanjing Medical University, Nanjing, China), weighing 300–325 g, were randomly divided into three groups (n=12 per group) as follows: Group A, the sham operation group; group B, the vehicle (PBS)-treated group and group C, the MSC-treated group. The rats were maintained under the following conditions: 24°C with 50% humidity; 7 am –7 pm light; 40–60 g/day food (18% protein, 4% fat, 5% fiber, 8% ash, 10% water) and 50–70 ml/day water. Surgical procedures were performed as described previously (21). Briefly, the 24 animals in groups B and C underwent open surgery under 2% sodium pentobarbital anesthesia (0.2 ml/100 g) on their right knees, involving anterior cruciate ligament transection and medial meniscectomy via an incision on the medial aspect of the joint capsule, anterior to the medial collateral ligament. The animals in group A underwent a sham operation on their right knees, in which a similar incision was made but the anterior cruciate ligament transection and medial meniscectomy were not performed. Following surgery, all the rats were intramuscularly administered antibiotics for three days and were allowed free activity without immobilization. Eight weeks post-surgery, the animals of group C received an intra-articular injection of 0.3 ml allogeneic MSCs (~5.0×10^5^ cells) into the right knee, while 0.3 ml PBS was injected into the right knees of groups A and B as a control. All rats were sacrificed by cervical dislocation at 14 weeks post-surgery. Half of the rats in each group were used for histology and the remainder were used for western blot analysis.

The femoral condyles were retrieved and fixed in 10% neutral-buffered formalin (Shenzhen Ketian Plastic Co., Ltd., Shenzhen, China) at 4°C for 48 h, then decalcified with EDTA (Sigma-Aldrich, St. Louis, MO, USA) for three weeks. The decalcified specimens were subsequently dehydrated in alcohol (Shenzhen Ketian Plastic Co., Ltd.), embedded in paraffin blocks (Shenzhen Ketian Plastic Co., Ltd.) and cut into 5-*µ*m sections. Serial sections, including the severely degenerated area, were stained with hematoxylin and eosin (HE), Safranin-O and toluidine blue (TB) (All from Sigma-Aldrich) using standard methods (http://protocolsonline.com/histology/dyes-and-stains/haematoxylin-eosin-he-staining/; https://www.medialabinc.net/spg531612/). The degree of cartilage degradation was scored using the modified Mankin score system (22). Histological analysis was performed by two blinded independent researchers.

The femoral condyle articular cartilage was pulverized into powder in liquid nitrogen (Nuotaishige, Nanjing, China), then lysis buffer (Sigma-Aldrich) was added and the mixture was centrifuged at 16,000 × g for 10 min at 4°C. Total protein extracted from the cartilage of the three groups was assessed by western blot.

### Western blot analysis

The proteins from chondrocytes and cartilage were isolated using the Total Protein Extraction kit (Beyotime Institute of Biotechnology) according to the manufacturer’s instructions. Harvested protein was subjected to SDS-PAGE and transferred onto polyvinylidene difluoride membranes (Invitrogen Life Technologies, Carlsbad, CA, USA). Following blocking in Tris-buffered saline (TBS; pH 7.6) containing 5% non-fat dried milk and 0.1% Tween-20, the membranes were probed with the following antibodies against: Col2 (1:100-1:200; ab185430; Abcam, Cambridge, MA, USA), rabbit polyclonal aggrecan (1:1,000; ab36861; Abcam), rabbit polyclonal COX-2 (1:300-1:1,000; ab52234; Abcam), rabbit polyclonal MMP-13 (1:3,000-1:6,000; ab39012; Abcam), mouse monoclonal β-actin (1:5,000-1:16,000; ab6276; Abcam), mouse monoclonal p-ERK1/2 (1:2,000; #9106; Cell Signaling Technology, Inc.), mouse monoclonal ERK1/2 (1:1,000; #9107; Cell Signaling Technology, Inc., Danvers, MA, USA), human polyclonal p-JNK (1:1,000; #9251; Cell Signaling Technology, Inc.), human polyclonal JNK (1:1,000; #9252; Cell Signaling Technology, Inc.), p-p38 (1:2,000; #9216; Cell Signaling Technology, Inc.), rabbit monoclonal p38 (1:1,000; #8690; Cell Signaling Technology, Inc.), mouse monoclonal p-NF-κB p65 (1:1,000; #13346; Cell Signaling Technology, Inc.), rabbit monoclonal NF-κB p65 (1:1,000; #8242; Cell Signaling Technology, Inc.) or mouse monoclonal IκBα (1:1,000; #4814; Cell Signaling Technology, Inc.) in blocking buffer and incubated overnight at 4°C. The membranes were subsequently washed three times with TBS and 0.1% Tween-20, incubated at 25°C for 2 h with goat anti-mouse (#170-6516), goat anti-rabbit (#170-6515) and goat anti-human (#172-1033) horseradish peroxidase-conjugated IgG secondary antibodies (1:5,000; Bio-Rad Laboratories, Inc., Hercules, CA, USA) and probed using a Super Signal West Pico chemiluminescent substrate kit (Pierce Biotechnology, Rockford, IL, USA). The membranes were scanned using a GS800 Densitometer Scanner (Bio-Rad Laboratories, Inc.), followed by data analysis using PDQuest 7.2.0 software (Bio-Rad Laboratories, Inc.).

### RNA extraction and RT-qPCR

Total RNA was extracted from chondrocytes using 1 ml TRIzol reagent (Invitrogen Life Technologies) according to the manufacturer’s instructions, dissolved in 0.1% diethylpyrocarbonate water and quantified by spectrophotometry at 260 nm absorbance using a Nucleic Quantitative Instrument (BioPhotometer plus; Eppendorf, Hamburg, Germany). A total of 1 *µ*g RNA was used to synthesize complementary DNA (cDNA) by reverse transcription using a PrimeScript™ RT reagent kit (Takara Bio, Inc., Otsu, Japan) according to the manufacturer’s instructions. Subsequently, the samples were subjected to qPCR using an ABI Prism 7500 detection system (Applied Biosystems Life Technologies, Foster City, CA, USA). Reactions in triplicate were conducted in 20 *µ*l reaction volume containing 1 *µ*l cDNA, 10 *µ*l Power SYBR Green PCR Master Mix (Applied Biosystems Life Technologies) and 250 nM of each primer. The primers were designed by Takara Biotechnology Co., Ltd (Dailan, China). The gene for GAPDH acted as an endogenous reference for normalization of fluorescence thresholds (Ct) values of target genes. PCR was performed using specific primers designed from the published sequence of each cDNA as follows: GAPDH sense, 5′-GGTGGACCTCATGGCCTACAT-3′ and antisense, 5′-GCCTCTCTCTTGCTCTCAGTATCCT-3′; Col2 sense, 5′-ACGCTCAAGTCGCTGAACAA-3′ and antisense, 5′-TCAATCCAGTAGTCTCCGCTCT-3′; aggrecan sense, 5′-TCCAAACCAACCCGACAAT-3′ and antisense, 5′-TCTCATAGCGATCTTTCTTCTGC-3′; MMP-13, sense, 5′-TGGTCCCTGCCCCTTCCCT-3′ and antisense, 5′-CCGCAAGAGTCACAGGATGGTAGT-3′; COX-2 sense, 5′-CCATCCTCCTTGAACACGG-3′ and antisense, 5′-TGCCACTGCTTGTACAGCG-3′. Messenger RNA expression was quantified according to the 2^−ΔΔCt^ method (23).

### Statistical analysis

Data are presented as the mean ± standard deviation. One-way analysis of variance was used to assess the significance of differences observed between groups. P<0.05 was considered to indicate a statistically significant difference. All data were analyzed using SPSS 16.0 software (SPSS Inc., Chicago, IL, USA).

## Results

### Characterization of bone marrow-derived MSCs

**Figure 1:**
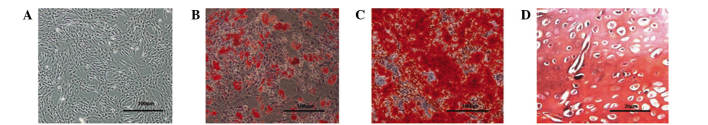
Characterization of MSCs. (A) The MSCs assumed a polymorphic, fibroblast-like morphology and were able to be differentiated into (B) adipocytes, following two weeks of culture in adipogenic medium (Oil red O staining), (C) osteoblasts, following three weeks of culture in osteogenic induction medium (Alizarin red staining) and (D) chondrocytes, following two weeks of culture in poly(lactic-co-glycolic acid) (Safranin O staining). The scale bars for A–C represent 100 *µ*m, while the scale bar in D represents 20 *µ*m. MSCs, mesenchymal stem cells.

To confirm that the cells isolated from rat bone marrow (Fig. 1A) were MSCs, their potential for multilineage differentiation was evaluated (Fig. 1B–D). Following 14 days of culture in adipogenic induction medium, the cells contained a large number of neutral lipid vacuoles (lipid droplets) that were positive for Oil Red O staining. Following three weeks of culture, the cells grown in osteogenic induction medium were positive for Alizarin red staining. The cartilaginous phenotype of the induced cells was confirmed by Safranin O staining following 14 days of culture. The ability of these cells to successfully differentiate into distinct cell types verified their status as MSCs.

### MSCs alter the gene and protein expression profiles of rat chondrocytes

**Figure 2:**
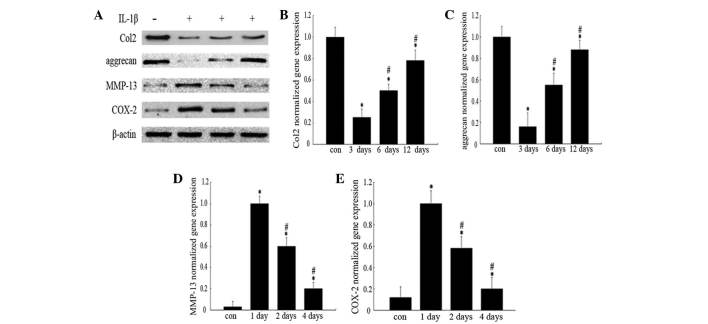
Effects of IL-1β on protein and gene expression levels. The groups represent chondrocytes without treatment (con) and chondrocytes cultured with normal medium for either 3, 6 or 12 days (for analysis of Col2 and aggrecan) or for 1, 2 or 4 days (for COX-2 and MMP-13 analysis) following incubation with 10 ng/ml IL-1β for 24 h, respectively. (A) Protein expression was analyzed by western blotting and the blot shown is representative of typical results (three repeats completed). Gene expression levels of (B) Col2, (C) aggrecan, (D) COX-2 and (E) MMP-13 were analyzed by RT-qPCR. Histograms represent the mean of values relative to the maximum value. RT-qPCR analyses were run in triplicate and the results are presented as the mean ± standard deviation. ^*^P<0.05 vs. control group; ^#^P<0.05 vs. 3/1 day group. IL-1β, interleukin-1β; Col2, type II collagen; COX-2, cyclooxygenase-2; MMP-13, matrix metalloproteinase-13; RT-qPCR, reverse transcription-quantitative polymerase chain reaction.

**Figure 3:**
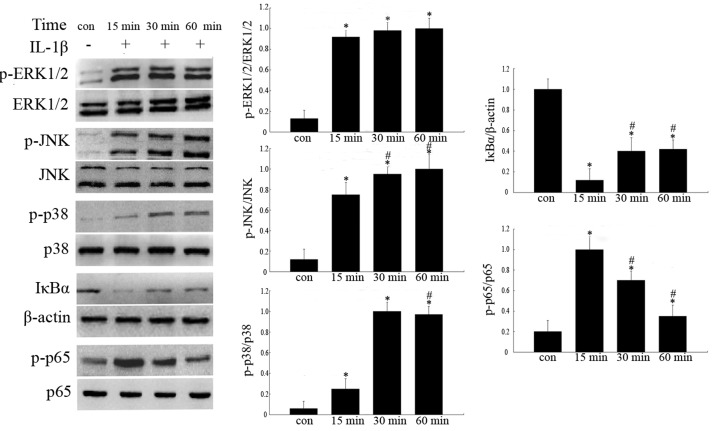
Analysis of expression levels of IL-1β-induced MAPK and NF-κB pathway-associated proteins. The four groups represent chondrocytes without treatment (con) and chondrocytes cultured with 10 ng/ml IL-1β for 15, 30 and 60 min, respectively. The proteins were analyzed by western blotting and quantified by densitometry. Histograms represent the mean values relative to the maximum value. Experiments were performed in triplicate and the bands shown represent typical results. Data are presented as the mean ± standard deviation. ^*^P<0.05 vs. control group; ^#^P<0.05 vs. 15 min group. IL-1β, interleukin-1β; MAPK, mitogen-activated protein kinase; NF-κB, nuclear factor κB, p-, phosphorylated; ERK1/2, extracellular signal-regulated kinases 1/2; JNK, c-Jun N-terminal kinase; IκBα, inhibitory-κ-B-α.

Western blot analysis revealed that the levels of Col2 and aggrecan protein (Fig. 2A) in IL-1β-treated chondrocytes were markedly lower than those in normal cells at the indicated time-points, while COX-2 and MMP-13 (Fig. 2A) expression levels were significantly higher than those in normal chondrocytes. The gene expression levels of Col2, aggrecan, MMP-13 and COX-2 in chondrocytes were analogous to those of the protein expression levels observed with western blot analysis (Fig. 2B–E). In addition, IκBα protein expression levels were markedly lower than those in normal chondrocytes, while, phosphorylated ERK1/2 (p-ERK1/2), p-JNK, p-p38 and p-p65 protein expression levels were significantly higher than those of normal chondrocytes (Fig. 3).

**Figure 4:**
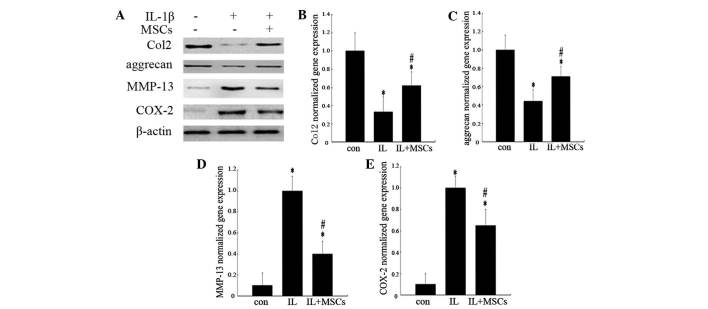
Effects of MSCs on protein and gene expression levels in chondrocytes incubated with IL-1β. The three groups represent chondrocytes without treatment (con) and chondrocytes co-cultured with (IL+MSCs) or without (IL) MSCs for three days (for analysis of Col2 and aggrecan) or for one day (for COX-2 and MMP-13 analysis) following incubation with 10 ng/ml IL-1β for 24 h, respectively. (A) Protein expression was analyzed by western blotting and the bands shown are representative of typical results. Gene expression levels of (B) Col2, (C) aggrecan, (D) MMP-13 and (E) COX-2 were analyzed by RT-qPCR. Histograms represent the mean of values relative to the maximum value. RT-qPCR analyses were run in triplicate and the results are presented as the mean ± standard deviation. ^*^P<0.05 vs. control group; ^#^P<0.05 vs. IL group. MSCs, mesenchymal stem cells; IL-1β, interleukin-1β; Col2, type II collagen; COX-2, cyclooxygenase-2; MMP-13, matrix metalloproteinase-13.

In order to evaluate whether factors secreted by MSCs effected inflammatory and catabolic processes in rat chondrocytes, chondrocytes were co-cultured with MSCs in a Transwell membrane system. As shown in Fig. 4A, co-culture of IL-1β-treated chondrocytes with MSCs resulted in a reduction in MMP-13 and COX-2 expression, compared with that of chondrocytes stimulated with IL-1β alone. By contrast, the protein expression levels of Col2 and aggrecan in chondrocytes co-cultured with MSCs were higher than those in chondrocytes treated with IL-1β alone. Changes to the gene expression levels of Col2, aggrecan, MMP-13 and COX-2 in chondrocytes were analogous to the results observed in the western blot analysis (Fig. 4B–E).

**Figure 5:**
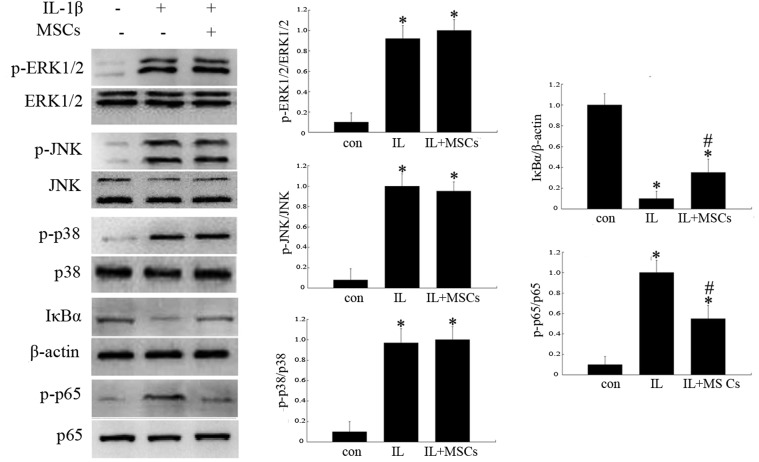
Effects of MSCs on the expression of MAPK and NF-κB pathway-associated proteins in rat chondrocytes incubated with IL-1β. The groups represent chondrocytes without treatment (con) and chondrocytes cultured with (IL+MSCs) or without (IL) MSC-conditioned medium for 15 min (for analysis of IκBα and NF-κB p65) or 30 min (for ERK1/2, JNK and p38 analysis) following incubation with 10 ng/ml IL-1β for 24 h, respectively. Protein expression was analyzed by western blotting and quantified by densitometry. Histograms represent the mean of values relative to the maximum value. Experiments were performed in triplicate and the bands shown represent typical results. Data are presented as the mean ± standard deviation. ^*^P<0.05 vs. control group; ^#^P<0.05 vs. IL group. IL-1β, interleukin-1β; MAPK, mitogen-activated protein kinase; NF-κB, nuclear factor κB, p-, phosphorylated; ERK1/2, extracellular signal-regulated kinases 1/2; JNK, c-Jun N-terminal kinase; IκBα, inhibitory-κ-B-α.

In order to evaluate potential signaling pathways influenced by MSCs, the expression of p-ERK1/2, p-JNK, p-p38, p-p65 and IκBα proteins were analyzed by western blotting. MSC-conditioned medium did not influence the phosphorylation of ERK1/2, JNK or p38 MAPK at the indicated time-points (Fig. 5); however, treatment with MSC-conditioned medium reduced the expression of p-p65 and increased the levels of IκBα (Fig. 5).

### MSC treatment alters the morphology and protein expression of cartilage

**Figure 6:**
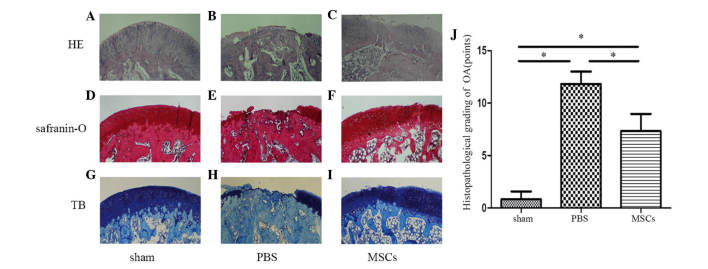
Histological analysis of cartilage. Photomicrographs showing serial sections (5 *µ*m) stained with (A–C) HE, (D–F) Safranin-O and (G–I) TB (magnification, ×40). (J) Histological grade of knee osteoarthritis assessed by modified Mankin criteria. Data are expressed as the mean ± standard deviation of six rats in each group. ^*^P<0.05. HE, hematoxylin and eosin; TB, toluidine blue; PBS, phosphate-buffered saline; MSCs, mesenchymal stem cells; OA, osteoarthritis.

**Figure 7:**
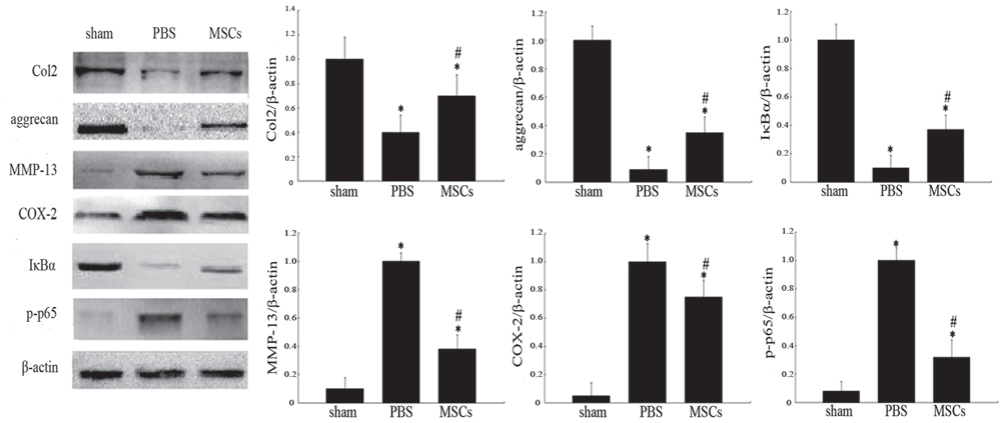
Protein expression levels in articular cartilage from femoral condyles of various groups. The three groups consist of the sham operation group (sham), vehicle-treated group (PBS) and MSC-treated group (MSCs). The proteins were analyzed by western blotting and quantified by densitometry. Histograms represent the mean of values relative to maximum value. Data are expressed as the mean ± standard deviation of six rats in each group. ^*^P<0.05 vs. sham group; ^#^P<0.05 vs. PBS group. PBS, phosphate-buffered saline; MSC, mesenchymal stem cell; Col2, type II collagen; COX-2, cyclooxygenase-2; MMP-13, matrix metalloproteinase-13; IκBα, inhibitory-κ-B-α; p-, phosphorylated.

Six weeks following the injection of MSCs, femoral condylar cartilage samples from the three groups were stained with HE, Safranin-O and TB and analyzed by western blotting for Col2, aggrecan, MMP-13, COX-2, IκBα and p-p65 (Fig. 6A–I). In the sham operation group, the articular cartilage was smooth, with intact superficial, mid and deep zones. The matrix surrounding the chondrocytes was arranged in columns which were smooth and evenly stained (Fig. 6A, D and G). The joints that were injected with PBS exhibited cartilage loss, almost exposing the bony layer. There was almost complete denudation of the articular cartilage with loss of the extracellular matrix, and no evidence of regeneration (Fig. 6B, E and H). The MSC-treated group exhibited reasonable cartilage regeneration compared with that of the PBS-treated group, but still exhibited surface discontinuity, including shallow vertical fissures through the cartilage superficial zone at numerous points across the surface and delamination of the superficial zone (Fig. 6C, F and I). At six weeks post-transplantation, significant differences were identified between the Mankin scores of the three groups (Fig. 6J). Western blot analysis revealed higher levels of Col2, aggrecan and IκBα expression in the MSC-treated group compared with that of PBS-treated group (Fig. 7). Furthermore, the bands of MMP-13, COX-2 and p-p65 were weaker in the MSC-treated group than those of the PBS-treated group. The results demonstrated that injection of MSCs downregulated MMP-13, COX-2 and p-p65 protein expression in osteoarthritic cartilage relative to the PBS group (Fig. 7).

## Discussion

Since Murphy *et al* (5) revealed that adult MSCs retard progressive cartilage destruction in a sheep OA model, MSC therapy has exhibited extensive potential for the treatment of OA (6–9). In the present study, it was demonstrated that MSCs promoted Col2 and aggrecan synthesis and reduced the inflammatory response in IL-1β-treated chondrocytes and a rat OA model. Previously, studies have focused on investigating the promotion of tissue repair by factors synthesized and secreted by MSCs (22,24–27). These trophic effects are distinct from the direct differentiation of MSCs into repair tissue, and have numerous advantages in regenerative medicine, including reducing the time and cost of cell amplification *in vitro* (28). Zuo *et al* (28) reported that the protein expression levels of Col2 and aggrecan were significantly upregulated in MSCs and chondrocytes co-cultured with or without direct cell-cell contact, compared with those of chondrocytes or MSCs cultured alone. The results of the present study confirmed that IL-1β increased COX-2 and MMP-13 expression and reduced Col2 and aggrecan expression. In addition, the present study aimed to investigate whether MSCs exerted chondroprotective effects via inhibition of COX-2 and MMP-13 in IL-1β-induced rat chondrocytes. As expected, the chondrocytes co-cultured indirectly with MSCs exhibited reduced expression of COX-2 and MMP-13 and upregulated expression of Col2 and aggrecan.

IL-1β is able to activate runt-related transcription factor 2, activator protein 1 and c-Maf, factors which significantly promote MMP-13 and COX-2 transcription, via the MAPK and NF-κB signaling pathways (13–15). The MAPK signaling pathways transduces numerous external signals, leading to a variety of cellular responses, including growth, differentiation, inflammation and apoptosis (29). The three subgroups of the MAPK family, the ERKs, JNKs and p38-MAPKs are structurally similar and have key roles in transmitting signals from the cell surface to the nucleus. NF-κB is retained in the cytoplasm during IκBα inactivity, while IL-1β activates NF-κB by triggering IκBα degradation. NF-κB activation results in the upregulation of a group of responsive genes that contribute to inflammation (30). Consequently, the present study aimed to investigate whether the MAPK or NF-κB pathways were involved in the expression of COX-2 and MMP-13 in IL-1β-treated chondrocytes cultured with MSC-conditioned medium. The results indicated that IL-1β upregulated the phosphorylation of ERK1/2, JNK, p38 and NF-κB p65 and downregulated the expression of IκBα. MSC-conditioned medium did not influence ERK1/2, JNK and p38 MAPK phosphorylation, but increased the levels of IκBα and reduced p-p65. Taken together, these results indicated that MSCs inhibit NF-κB activation in IL-1β-induced chondrocytes. It was therefore hypothesized that the inhibitory effect of MSCs on COX-2 and MMP-13 expression was, to some extent, attributable to their inhibition of the NF-κB pathway. Owing to the limited incubation times investigated in the present study, the possibility that the inhibitory effect of MSCs may occur via the MAPK pathway was not excluded entirely.

The identification of which specific factors secreted by MSCs contribute to the anti-inflammatory effect observed remains to be elucidated. Proteins secreted by MSCs of mouse and human origin have been analyzed by a variety of methods, and found to include chemokines, cytokines, growth factors and protease inhibitors (24). Many of these, including IL-4, -10 and -13, transforming growth factor-β (TGF-β), macrophage migration inhibitory factor, leukocyte migration inhibitory factor and metalloproteinase inhibitors, possess the ability to inhibit the release of inflammatory molecules (31). The pro-inflammatory cytokines and other signals expressed by injured cells induce MSCs to secrete anti-inflammatory factors, including tumor necrosis factor-α stimulated gene/protein 6, prostaglandin E2 and IL-1 receptor antagonist, that mediate the activation of resident macrophages or decrease the downstream effects of pro-inflammatory cytokines. The net effect is to decrease the activation of NF-κB in resident macrophages by parenchymal cells, via the secretion of IL-6, chemokine (C-X-C motif) ligand 1 and associated factors, and to decrease the recruitment of neutrophils (32). In a previous study by our group (unpublished), the expression of TGF-β by MSCs was blocked by transfection with slow virus and the results revealed that the expression of Col2 and aggrecan in co-cultured chondrocytes was decreased compared with those co-cultured with normal MSCs, which suggested a significant association between TGF-β and the regeneration of injured cartilage. There are numerous bioactive factors, but the identification of which of these are involved in inhibiting the degeneration of chondrocytes remains to be elucidated. Further studies are required to clarify the specific mechanism underlying the modulation of MMP-13 and COX-2 by MSCs.

In conclusion, the results of the present study demonstrated that MSCs suppress the IL-1β-induced Col2 and aggrecan degeneration, as well as the expression of MMP-13 and COX-2 in rat chondrocytes and in the cartilage of a rat osteoarthritic model, in part via the NF-κB signaling pathway.
